# Prevalence of Endodontically Treated Premolars and Molars With Untreated Canals and Their Association With Apical Periodontitis Using Cone-Beam Computed Tomography

**DOI:** 10.7759/cureus.25619

**Published:** 2022-06-03

**Authors:** Yousef Alnowailaty, Faisal Alghamdi

**Affiliations:** 1 Conservative Dentistry, Faculty of Dentistry, King Abdulaziz University, Jeddah, SAU; 2 Oral Biology, Faculty of Dentistry, King Abdulaziz University, Jeddah, SAU

**Keywords:** cone-beam computed tomography, apical periodontitis, missed canal, prevalence, saudi population

## Abstract

Background

This retrospective study evaluated the prevalence of untreated canals in root-canal-treated maxillary and mandibular posterior teeth and their association with apical periodontitis (AP) in a Saudi Arabian population. This study is based on a radiographic examination of scans taken using cone-beam computed tomography (CBCT).

Methodology

The study comprised CBCT scans obtained from 300 individuals (150 women and 150 men) aged 18 to 80 years. Images were evaluated for the presence of AP related to untreated canals of endodontically treated maxillary and mandibular posterior teeth. Disruption in the lamina dura surrounding the breadth of periodontal ligaments at the apical third of the roots was described as a periapical lesion. The outcomes were presented in the form of frequencies and percentages. To assess proportional differences, the chi-square test was performed, with the significance level set at ≤0.05.

Results

The overall percentage of untreated canals among endodontically treated teeth was 12.46%. The prevalence of untreated canals was the highest in maxillary second molars (38.1%) (p = 0.045). The prevalence of AP among teeth with untreated canals was 85.4%, with 88.5% in the maxilla (p = 0.0347) and 81.8% in the mandible (p = 0.010).

Conclusions

The prevalence of AP in root-canal-treated teeth with missed canals was high (85.4%), with most identified untreated canals in maxillary and mandibular first molars.

## Introduction

The primary goal of root canal treatment (RCT) is to conduct proper biomechanical cleaning, shaping, and filling of the entire root canal system in a three-dimensional (3D) manner. Failure to do so might lead to negative outcomes [1]. There are several causes of endodontic failure, including chronic bacterial infection [2], poor root filling [3], and missed/untreated canals [4]. Investigations have revealed that RCT failure is associated with untreated canals ranging between 12% and 42% in various demographics [5-8]. Several studies have discussed the importance of locating, cleaning, and filling all existing canals within the root canal system for optimal prognosis. Furthermore, the potential negative effect of untreated canals on outcomes has been evaluated, with evidence of a high prevalence of missed canals in failed cases requiring endodontic retreatment [8-10].

According to a previously reported study, apical periodontitis (AP) is significantly higher (98%) in endodontically treated teeth with missed canals than in those with no missed canals [6]. A lack of understanding of the root canal system and its intricacies can lead to canals being missed during RCT. These canals may harbor microbes and have a detrimental effect on the prognosis [11-13].

A conventional intraoral radiograph has been utilized in previous investigations [14-17] to assess the post-treatment AP. Nevertheless, the inherent limitations of the two-dimensional (2D) evaluation method have made locating untreated canals challenging. On the contrary, the recently introduced cone-beam computed tomography (CBCT) imaging allows for exact 3D viewing of a specific tooth [7].

The incidence of untreated canals in endodontically treated teeth and its correlation with apical pathologies has been assessed using CBCT in recent investigations involving different populations [5-7]. However, there is a lack of data within Middle Eastern countries, especially Saudi Arabia [6,7,18]. A recent investigation was conducted in Jazan, Saudi Arabia, utilizing CBCT to assess the prevalence of untreated canals and their association with AP [19]. This research sheds light on the importance of understanding root canal geometry before initiating endodontic therapy, as well as identifying the most likely canals to be missed and treating them properly. Therefore, this study aimed to establish the prevalence of untreated canals in previously root-canal-treated posterior teeth and their association with AP within a Saudi adult population. Furthermore, this study evaluated the relationship between untreated canals and AP prevalence, gender, and tooth type (maxillary/mandibular tooth) using CBCT scans.

## Materials and methods

Selection of CBCT images

**Figure 1:**
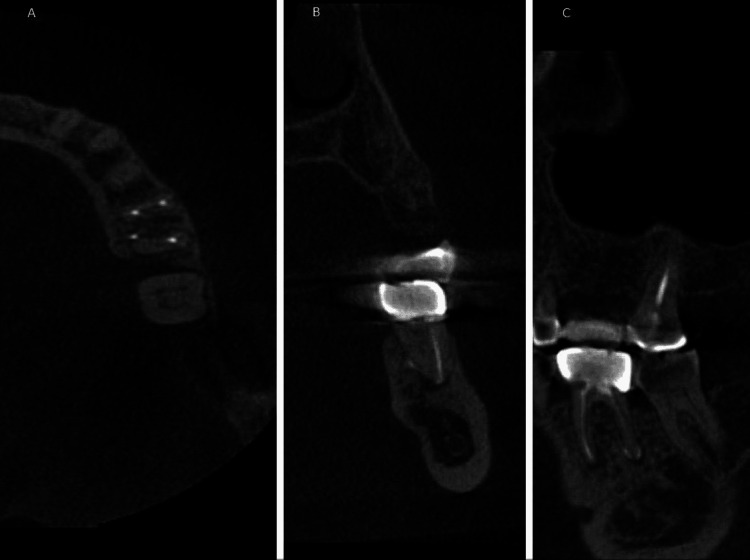
Cone-beam computed tomography of molar #36 with apical radiolucency on distolingual root because of the untreated distolingual canal. A: The axial plane; B: the coronal plane; C: the sagittal plane.

This research was conducted in the Oral and Maxillofacial Radiology Department of King Abdulaziz University (KAU) Dental Hospital (Jeddah, Saudi Arabia). We performed a random screening of 2,179 CBCT scans. Selected samples included Saudi citizens living in Jeddah with high-quality CBCT scans and at least one endodontically treated posterior tooth (Figure 1). Based on the American Society of Anesthesiologists (ASA) Physical Status Classification System [20], we included only healthy patients (ASA I) or those with mild systemic disease (ASA II). The excluded scans were either from Saudi residents (non-citizens), patients with other ASA classifications (III-VI), unclear/distorted CBCT images, lacking at least one endodontically treated posterior tooth, or were of teeth with immature apices, retained primary teeth, or remaining roots.

CBCT images were obtained for various reasons, including treatment planning in cases of implant surgery, tooth impaction, orthodontic therapy, or trauma, as well as in teeth with a complex/unusual root canal system before undergoing RCT. Demographic information including gender, country, and age was accessible for each scan following the ethics committee of the Faculty of Dentistry at KAU without exposing or sharing the protected health information of patients. In this investigation, a multi-stage stratified random sample with two database groups for CBCT scans was used. Based on the inclusion criteria, the CBCT scans of 300 individuals (150 women and 150 men) aged 18-80 years (mean ± standard deviation (SD) = 40.1 ± 18.1) collected between January 2018 and November 2021 were randomly selected from each database group and included in this study. All research subjects were Saudi Arabian citizens residing in Jeddah to rule out ethnic differences in the number or type of endodontically treated posterior maxillary and mandibular teeth with untreated canals. All Saudi Arabian citizens and residents who did not live in the city or those who lived in other provinces of Saudi Arabia were excluded from this investigation for reducing the ethnic differences and focusing on the city population.

The authors are accountable for ensuring the accuracy or integrity of this work. The study was conducted conforming to the ethical standards of the Declaration of Helsinki. The study was approved by the Research Ethics Committee of the Faculty of Dentistry, KAU (approval number: 291-10-21). Written informed consent of patients was not required for this retrospective analysis because all CBCT scans were reviewed retrospectively from the archives of the Oral and Maxillofacial Radiology Department, Faculty of Dentistry, KAU Dental Hospital.

Power analysis and sample size calculation

Study power was calculated by applying an independent t-test. For the provided values from the t-test with an alpha (α) level of 0.05 (5%), the power was 0.85, and the sample size was 1,000 (500 patients per group for both males and females). To estimate sample size, we used the Power and Sample Size Calculation version 3.1.6 (PS software, Vanderbilt University, Nashville, TN, USA).

Image acquisition

We utilized an i-CAT 1719 3D digital imaging system (Imaging Sciences International, Hatfield, PA, USA) with a standardized protocol and settings for CBCT image acquisition. We used the following settings: (i) 120 kV, 5-8 mA as the exposure setting; (ii) 17.5-26.9 seconds as the exposure duration; and (iii) a field of view (FOV) of 8 × 8 cm and voxel size of 0.125 × 0.125 × 0.125 mm because the voxel size was in 3D for the included recorded scans based on the inclusion criteria applied in this investigation.

Image evaluation

For image reconstruction and measurements, the OnDemand 3D Imaging Software (Cybermed, Seoul, South Korea) was used. The scans were recreated in the axial, coronal, and sagittal planes. In addition, multiplanar reconstruction (MPR) was used to obtain a comprehensive picture of the root canal morphology. The MPR was performed by going in the coronal-apical and apical-coronal directions. If the scan in one of the three planes was unclear, the procedure was repeated, and the tooth was 3D inspected (Figure 1).

The number of missed canals in root-canal-treated posterior teeth, the prevalence of AP lesions, and the prevalence of missed canals in the posterior teeth associated with AP were noted depending on tooth type (maxillary/mandibular tooth) in the same individual showing distinctive planes (axial, sagittal, coronal).

Two experienced readers of CBCT scans (YA and FA) examined the scans and determined the number of missed canals in endodontically treated posterior teeth with AP lesions. For calibration, both authors and one consultant radiologist reviewed 100 randomly selected CBCT images that were not involved in the investigation. It was conducted at two weekly intervals throughout the investigation. A consultant radiologist was needed to maintain the uniformity of CBCT scan assessment.

Periapical status

Based on radiographic criteria, the periapical state of the teeth was defined as unhealthy when there was a disruption of the lamina dura and the low-density region related to the radiographic apex was twice the breadth of the periodontal ligament gap [21,22]. In multi-rooted teeth, the canals with the poorest periapical state were combined to reflect the overall periapical status of the tooth. Teeth were considered to be healthy when the periodontal ligament gap was normal or slightly wider than usual, with no apparent bone rarefaction. AP was defined when periapical radiolucency, widening of periodontal ligament gap, and disturbed lamina dura was noted [21,22].

Statistical analysis

We used SPSS version 20.0 for Windows (IBM Corp., Armonk, NY, USA) to analyze the collected data. Cohen’s kappa test was applied to assess interexaminer and intraexaminer reliability for CBCT scan interpretation. The data are presented in the form of frequencies and percentages. An independent t-test was applied to calculate the power analysis of the sample size, while the chi-square test was used to assess the correlation between gender and tooth type (maxillary/mandibular tooth) regarding the number of untreated root canals in posterior teeth with AP. The statistical level of significance was set at p-values of 0.05.

## Results

Interexaminer and intraexaminer reliability

There was an excellent interexaminer agreement regarding the presence/absence of untreated canals (kappa ≥ 0.98), AP (kappa ≥ 0.97), and untreated canals associated with AP (kappa ≥ 0.96) (Table 1). Regarding intraexaminer reliability, there was excellent agreement between the two examiners regarding the presence/absence of untreated canals (kappa ≥ 0.97 and kappa ≥ 0.95), AP (kappa ≥ 0.96 and kappa ≥ 0.96), and untreated canals associated with AP (kappa ≥ 0.98 and kappa ≥ 0.98) (Table 2).

**Table 1:** Inter-examiner agreement values. a: not assuming the null hypothesis; b: using the asymptotic standard error assuming the null hypothesis.

Measure of agreement (kappa) for different variables	Kappa value	Asymptotic standard error^a^	Approximate T^b^	Approximate significance
Presence/absence of untreated canals	0.988	0.012	19.387	0.000
Presence/absence of apical periodontitis	0.976	0.017	19.152	0.000
Presence/absence of untreated canals associated with apical periodontitis	0.963	0.021	18.903	0.000
Number of valid cases	385			

**Table 2:** Intraexaminer agreement of examiner 1 versus examiner 2. a: not assuming the null hypothesis. b: using the asymptotic standard error assuming the null hypothesis.

Measure of agreement (kappa) for different variables	Kappa value	Asymptotic standard error^a^	Approximate T^b^	Approximate significance
Presence/absence of untreated canals (examiner 1)	0.976	0.017	19.152	0.000
Presence/absence of apical periodontitis (examiner 1)	0.963	0.021	18.903	0.000
Presence/absence of untreated canals associated with apical periodontitis (examiner 1)	0.988	0.012	19.383	0.000
Number of valid cases	385			
Presence/absence of untreated canals (examiner 2)	0.951	0.025	18.658	0.000
Presence/absence of apical periodontitis (examiner 2)	0.963	0.021	18.903	0.000
Presence/absence of untreated canals associated with apical periodontitis (examiner 2)	0.988	0.012	19.383	0.000
Number of valid cases	385			

Patient characteristics

All 300 patients were Saudi citizens, with 279 (93%) patients classified as ASA I and 21 (7%) as ASA II based on the ASA Physical Status Classification System [20]. Of the 21 patients with ASA II, 17 (81%) had well-controlled hypertension (HTN) and four (19%) had well-controlled diabetes mellitus (DM) (type 1 DM, 25% (n = 1); type 2 DM, 75% (n = 3)). Only patients classified as ASA II were taking pharmacotherapies for their medical conditions. For HTN patients, the medications included diuretics in six (35%) patients, beta-blockers in four (24%) patients, and angiotensin-converting enzyme (ACE) inhibitors in seven (41%) patients. DM medications included insulin (rapid-acting) in one (25%) patient, metformin (biguanide class) in two (50%) patient, and glipizide (sulfonylurea class) in one (25%) patient. No information regarding the immune status and periodontal status before endodontic therapy was available. However, the overall number of endodontically treated posterior teeth in the 300 CBCT scans was 385 teeth (maxillary teeth = 201: first premolars (n = 54), second premolars (n = 52), first molars (n = 53), and second molars (n = 42); mandibular teeth = 184: first premolars (n = 17), second premolars (n = 30), first molars (n = 92), and second molars (n = 45)).

Prevalence of untreated canals

The overall prevalence of untreated canals among endodontically treated posterior teeth was 12.46% (n = 48/385) (maxillary teeth = 26, mandibular teeth = 22) (Table 3). The prevalence of untreated canals was higher in the maxillary teeth than in the mandibular teeth (26 vs. 22; p = 0.012 in maxillary teeth, p = 0.005 in mandibular teeth). Overall, the highest prevalence of untreated canals was in the maxillary second molars (38.1%; p = 0.045), followed by the maxillary first molars (15.1%; p = 0.404). The lowest prevalence was found in the maxillary second premolars (3.8%; p = 0.502). There were no missed canals among the maxillary first premolars (Table 3).

**Table 3:** Prevalence of untreated teeth among maxillary and mandibular endodontically treated premolars and molars based on gender and type of tooth.

Type of endodontically treated teeth (n = 385)		Untreated canals				Total number of untreated canal cases (%)	Chi-square	P-value
		Males		Females				
		Yes (%)	No (%)	Yes (%)	No (%)			
Maxillary teeth (n = 201)	First premolars (n =54)	0 (0.0%)	10 (18.5%)	0 (0.0%)	44 (81.5%)	0 (0.0%)	--	--
	Second premolars (n = 52)	0 (0.0%)	15 (28.8%)	2 (3.8%)	35 (67.3%)	2 (3.8%)	0.843	0.502
	First molars (n = 53)	3 (5.7%)	12 (22.6%)	5 (9.4%)	33 (62.3%)	8 (15.1%)	0.393	0.404
	Second molars (n = 42)	10 (23.8%)	8 (19%)	6 (14.3%)	18 (42.9%)	16 (38.1%)	4.072	0.045
	Total (n = 201)	13 (6.5%)	45 (22.4%)	13 (6.5%)	130 (64.6%)	26 (12.9%)	6.503	0.012
Mandibular teeth (n = 184)	First premolars (n = 17)	0 (0.0%)	1 (5.9%)	0 (0.0%)	16 (94.1%)	0 (0.0%)	--	--
	Second premolars (n = 30)	0 (0.0%)	9 (30%)	0 (0.00%)	21 (70%)	0 (0.0%)	--	--
	First molars (n = 92)	9 (9.8%)	30 (32.6%)	5 (5.4%)	48 (52.2%)	14 (15.2%)	3.241	0.067
	Second molars (n = 45)	5 (11.1%)	12 (26.7%)	3 (6.7%)	25 (55.6%)	8 (17.8%)	2.530	0.118
	Total (n = 184)	14 (7.6%)	52 (28.3%)	8 (4.3%)	110 (59.8%)	22 (11.9%)	8.330	0.005

All untreated canals in the mandible were exclusive to molars with the following prevalence: first molars (15.2%; p = 0.067) and second molars (17.8%; p = 0.118) (Table 3). Even though there was a statistically significant relationship between total untreated canal cases and gender in maxillary teeth (p = 0.012) and mandibular teeth (p = 0.005), males (7.6%) had a greater frequency of untreated canals in mandibular posterior teeth when compared to females (4.3%) (Table 3). The second mesiobuccal (MB2) canal was the most likely to be missed with/without AP in the maxillary first molars with eight (100%) teeth and second molars with 14 (87.5%) teeth, followed by the palatal canal with two (100%) teeth in maxillary second premolars and the distobuccal canal with two (12.5%) teeth in maxillary second molars (Table 4). In the mandible, the most untreated canals with/without AP included the mesiolingual canal in the first molars with five out of 14 teeth (35.7%), the mesiobuccal canal in the first molars with seven out of 14 teeth (50%), and the second molars with eight out of eight teeth (100%). We only detected one case of a missed distolingual canal (7.1%) and one case of a missed distal canal (7.1%) in mandibular first molars (Table 5).

**Table 4:** Distribution of the untreated canals with apical periodontitis in maxillary teeth based on gender and type of tooth.

Type of untreated teeth		Canal	Apical periodontitis				Total number of untreated canals with apical periodontitis cases (%)	Chi-square	P-value
			Males		Females				
			Yes (%)	No (%)	Yes (%)	No (%)			
Maxillary teeth (n = 26)	Second premolars (n = 2)	Buccal	0 (0.00%)	0 (0.00%)	0 (0.00%)	0 (0.00%)	0 (0.00%)	-	-
		Palatal	0 (0.00%)	0 (0.00%)	2 (100%)	0 (0.00%)	2 (100%)	-	-
	First molars (n = 8)	Mesiobuccal	3 (37.5%)	0 (0.00%)	5 (62.5%)	0 (0.00%)	8 (100%)	-	-
		Distobuccal	0 (0.00%)	0 (0.00%)	0 (0.00%)	0 (0.00%)	0 (0.00%)	-	-
		Palatal	0 (0.00%)	0 (0.00%)	0 (0.00%)	0 (0.00%)	0 (0.00%)	-	-
	Second molars (n = 16)	Mesiobuccal	7 (43.8%)	2 (12.5%)	4 (25%)	1 (6.3%)	11 (68.8%)	0.162	0.922
		Distobuccal	1 (6.3%)	0 (0.00%)	1 (6.3%)	0 (0.00%)	2 (12.5%)	0.152	0.625
		Palatal	0 (0.00%)	0 (0.00%)	0 (0.00%)	0 (0.00%)	0 (0.00%)	-	-
	Total (n = 26)	All canals	11 (42.3%)	2 (7.7%)	12 (46.2%)	1 (3.8%)	23 (88.5%)	2.120	0.347

**Table 5:** Distribution of the untreated canals with apical periodontitis in mandibular teeth based on gender and type of tooth.

Type of untreated teeth		Canal	Apical periodontitis				Total number of untreated canals with apical periodontitis cases (%)	Chi-square	P-value
			Males		Females				
			Yes (%)	No (%)	Yes (%)	No (%)			
Mandibular teeth (n = 22)	First molars (n = 14)	Mesiobuccal	5 (35.7%)	0 (0.00%)	0 (0.00%)	2 (14.3%)	5 (35.7%)	6.533	0.038
		Mesiolingual	3 (21.4%)	0 (0.00%)	0 (0.00%)	2 (14.3%)	3 (21.4%)	5.289	0.071
		Distobuccal	0 (0.00%)	0 (0.00%)	0 (0.00%)	0 (0.00%)	0 (0.00%)	-	-
		Distolingual	0 (0.00%)	0 (0.00%)	1 (7.1%)	0 (0.00%)	1 (7.1%)	1.938	0.357
		Single mesial canal	0 (0.00%)	0 (0.00%)	0 (0.00%)	0 (0.00%)	0 (0.00%)	-	-
		Single distal canal	1 (7.1%)	0 (0.00%)	0 (0.00%)	0 (0.00%)	1 (7.1%)	0.598	0.643
	Second molars (n = 8)	Mesiobuccal	5 (62.5%)	0 (0.00%)	3 (37.5%)	0 (0.00%)	8 (100%)	-	-
	Total (n = 22)	All canals	14 (63.6%)	0 (0.00%)	4 (18.2%)	4 (18.2%)	18 (81.8%)	8.556	0.010

Prevalence of apical periodontitis

The overall prevalence of AP among 300 CBCT images of endodontically treated posterior teeth was 15.84% (n = 61/385; maxillary teeth = 35, mandibular teeth = 26) (Table 6). The prevalence of AP was higher in the maxillary teeth (17.4%; p = 0.005) than in the mandibular teeth (14.1%; p = 0.001) (Table 6). There was only a slightly higher incidence of AP in maxillary posterior teeth among females (9%) when compared to males (8.5%) (Table 6). In the mandibular teeth, there was a significantly higher prevalence of AP among males (9.2%) than females (4.9%). There was a statistically significant observation regarding the prevalence of AP among the maxillary second molars (p = 0.039) and among mandibular first molars (p = 0.039). There was no statistical significance reported for other posterior teeth; however, AP incidence was more common in males (Table 6).

**Table 6:** Prevalence of apical periodontitis among maxillary and mandibular endodontically treated premolars and molars based on gender and type of tooth.

Type of endodontically treated teeth (n = 385)		Apical periodontitis				Total number of apical periodontitis cases (%)	Chi-square	P-value
		Males		Females				
		Yes (%)	No (%)	Yes (%)	No (%)			
Maxillary teeth (n = 201)	First premolars (n = 54)	1 (1.9%)	9 (16.7%)	3 (5.5%)	41 (75.9%)	4 (7.4%)	0.120	0.571
	Second premolars (n = 52)	1 (1.9%)	14 (26.9%)	2 (3.8%)	35 (67.3%)	3 (5.8%)	0.031	0.648
	First molars (n = 53)	4 (7.5%)	11 (20.8%)	6 (11.3%)	32 (60.4%)	10 (18.9%)	0.831	0.293
	Second molars (n = 42)	11 (26.2%)	7 (16.7%)	7 (16.7%)	17 (40.5%)	18 (42.9%)	4.286	0.039
	Total (n = 201)	17 (8.5%)	41 (20.4%)	18 (9%)	125 (62.2%)	35 (17.4%)	8.024	0.005
Mandibular teeth (n = 184)	First premolars (n = 17)	0 (0.00%)	1 (5.9%)	0 (0.0%)	16 (94.1%)	0 (0.0%)	--	--
	Second premolars (n = 30)	2 (6.7%)	7 (30%)	1 (3.3%)	20 (66.6%)	3 (10.0%)	2.134	0.207
	First molars (n = 92)	10 (10.9%)	29 (31.5%)	5 (5.4%)	48 (52.2%)	15 (16.3%)	4.325	0.037
	Second molars (n = 45)	5 (11.1%)	12 (26.7%)	3 (6.7%)	25 (55.6%)	8 (17.8%)	2.530	0.118
	Total (n = 184)	17 (9.2%)	49 (26.6%)	9 (4.9%)	109 (59.2%)	26 (14.1%)	11.466	0.001

Prevalence of untreated canal associated with apical periodontitis

The overall prevalence of untreated canals associated with AP among endodontically treated posterior teeth in 300 CBCT images was 85.4% (n = 41/48; maxillary teeth = 23, mandibular teeth = 18) (Tables 4, 5). No statistical significance was noted in untreated canals associated with AP among the maxillary teeth (88.5%; p = 0.347); however, females had a slightly greater incidence of untreated canals with AP in maxillary posterior teeth (46.2%) than males (42.3%) (Table 4). On the contrary, there was a statistically significant number of missed canals associated with AP observed in the mandibular teeth (81.8%; p = 0.010); however, untreated canals with AP were more prevalent in males (Table 5). The MB2 canal in the maxillary first molars and maxillary second molars were most often missed associated with AP (100% and 68.8%, respectively; p = 0.922) (Table 4). In the mandible, the mesiobuccal and mesioligual canals in the mandibular first molars were the most untreated canals associated with AP by 35.7% (p = 0.038) and 21.4% (p = 0.071), respectively, followed by the mesiobuccal canal in the mandibular second molars (100%) (Table 5).

## Discussion

In this retrospective, cross-sectional study, the prevalence of untreated canals was assessed in endodontically treated maxillary and mandibular posterior teeth using CBCT scans in one hospital in Jeddah, Saudi Arabia. We have presented data on untreated canal prevalence during a specific period.

Devitalized infected pulp space may act as a microbial niche maintaining or causing a periradicular disease [23]. The oral bacteria infiltrate root canals following pulpal exposure [24]. The colonizing species can then proliferate and form adhesions to the root canal walls, and microorganisms may detach, gradually leading to the infection spreading toward the apex [25]. These inflammatory processes in the periapical periodontium cause AP [26]. A retrospective cohort study by Ruiz et al. found that the risk of AP in endodontically treated teeth is five times higher in patients with periodontal disease than in those without periodontal disease due to increased permeability of the dentinal tubules to periodontal pathogens [27]. Thus, a missed canal within endodontically treated teeth can lead to AP formation [11]. CBCT images are high-resolution 3D images, which eliminates many of the limitations of conventional radiographs [28]. Recently, CBCT has become increasingly popular in endodontic diagnosis and treatment planning [29]. The AAE/AAOMR recommends that limited FOV of CBCT should be considered first in the initial treatment of cases with potential extra canals or complex anatomy [30]. Huumonen et al. compared the diagnostic accuracy of CBCT to intraoral radiographs regarding retreatment decision-making surrounding upper molars. CBCT revealed that MB2 was unfilled in 90% of the samples where their presence was confirmed. Moreover, 80% of unfilled canals were associated with AP requiring retreatment [10]. A recent study has shown that using CBCT preoperatively as a treatment planning tool has a strong impact on the decision-making process [31].

There may be severe consequences if the missed canal is not treated. Bacteria residing in these untreated canals can lead to persistent or secondary infections [4,32,33]. Asymptomatic AP can become symptomatic AP or shift to an acute or chronic apical abscess [34,35]. In cases spreading beyond the dental alveolar boundary, the apical abscess can be confined to the periapical area or continue to diffuse through adjacent bones and soft tissues as a diffuse abscess or cellulitis [36]. When the apical abscess remains untreated, it can reach blood circulation and cause systemic complications [37].

An endodontic body of evidence shows that a significant number of untreated canals in root-canal-treated dentitions required non-surgical endodontic retreatment. Hoen and Pink [8] detected untreated canals in 42% of all non-surgically retreated teeth. Moreover, Karabucak et al. [7] reported a 23% overall frequency, while Mashyakhy et al. [19] found an 18% overall prevalence of untreated canals. Costa et al. [6] and Baruwa et al. [5] found a lower prevalence of 12%. The prevalence of untreated canals among endodontically treated teeth in this study was 12.46%. The difference between the current research and previously published data might be due to variations in the study sample, population, and methodology. Nevertheless, the outcomes revealed the same finding. In our investigation, 85.4% of RCT teeth with untreated canals had AP. Baruwa et al. [5] and Karabucak et al. [7] reported 82.6% and 82.8% of untreated canals associated with AP, respectively. Furthermore, Mashyakhy et al. [19] and Costa et al. [6] found a very high incidence of AP (90% and 98%, respectively) among teeth with untreated canals. All previous investigations [5-7,19] have illustrated a strong association between untreated canals and AP compared to the current findings.

Maxillary molars exhibit a complicated root canal system with two canals in the mesiobuccal root (the second one is known as MB2) [38-41]. Previous investigations have reported that the mesiobuccal root has the highest incidence of untreated canals at 62.8%, 85%, 65%, and 61%, respectively [5-7,19]. These findings are comparable to our findings where 22 (84.6%) of the mesiobuccal canals in maxillary molars had untreated canals (Table 4). In mandibular teeth, most untreated canals were the mesiobuccal ones in the seven first molars (31.8%), and eight second molars (36.4%). In contrast, a recent investigation on mandibular first molars revealed that 27.3% of untreated canals were in the mesiobuccal canals [19]. The increased incidence of untreated mesiobuccal canals in mandibular first molars in the current investigation might be attributed to the larger sample size compared to other studies (31.8% vs. 27.3%) [19].

Several factors can affect the results and affect endodontic treatment outcomes. In this study, the medical conditions and pharmacotherapies of the patients were considered as factors that might influence endodontic treatment outcomes. Other factors such as immune status and AP status before the endodontic therapy were not considered as they were not recorded in the dental files. Only 7% of the selected patients were classified as ASA II and had well-controlled HTN (17 patients) and well-controlled DM (four patients), while 93% of the selected patients were healthy (ASA I).

A 2010 study [42] found a correlation between AP and root-filled teeth in 13 (72%) patients who had HTN, which was not statistically different from that of the nine (45%) patients who did not have HTN. DM has been linked with severe gingivitis and periodontitis [43]. Thus, DM may be a predisposing factor for oral infections and a risk factor for AP, which manifests as a flare-up and may increase the failure rate of RCT. Two studies [44,45] verified DM to be a risk factor for developing AP. Moreover, in comparison with well-controlled DM, poor glycemic administration may be correlated with a higher prevalence of AP and a higher risk of endodontic treatment failure [46]. Tibúrcio-Machado et al. [47] concluded in a critical review that the findings were rudimentary and the evidence for such a correlation was not decisive. The published findings indicate a favorable correlation between DM and more periapical lesions [47].

In our study, among the 41 teeth that had untreated canals with AP, the distribution of frequencies and percentages was very low in ASA II patients as follows: six (35%) out of 17 teeth were from patients with well-controlled HTN, four (100%) out of four teeth were from patients with well-controlled DM, and 23 (85%) out of 27 teeth were from healthy patients (ASA I). Thus, only 10 (50%) out of 21 teeth were untreated canals with AP from patients with well-controlled HTN and DM. There were seven teeth that had untreated canals without AP, and three (43%) out of seven teeth were from patients with well-controlled HTN compared to four (57%) out of seven teeth from healthy patients. There were 21 (43.75%) out of 48 teeth that had untreated canals with/without AP in ASA II patients compared to 27 (56.25%) out of 48 teeth from healthy patients (ASA I). All of the 17 HTN and four DM patients in this study were well-controlled. Thus, these underlying medical conditions might not have a major effect on AP development. In a recent study [48], there was an increased risk of developing AP in endodontically treated teeth due to gingival status, coronal restoration quality, and root canal filling quality rather than the patient’s medical condition. Thus, this may be an RCT failure.

Therefore, our analysis of these missed canals among endodontically treated teeth has expanded the knowledge base of AP diagnosed by CBCT. Moreover, several factors might influence endodontic treatment outcomes that were not considered in this study, such as the sealer material used, the material of the core, and the root canal sealing technique. However, dentists performing RCT should evaluate the prevalence rate of AP in such cases by tooth location, periodontal status before endodontic treatment, type of prosthesis, materials of core, RCT method, materials of sealer, and endodontic canal retreatment.

Study strengths and limitations

This research evaluated all available CBCT scans of endodontically treated posterior teeth that met the inclusion criteria. This is the first retrospective study with a cross-sectional design to address untreated canals associated with AP prevalence among Saudi Arabians in Jeddah. The study included 300 CBCT scans obtained between 2018 and 2021. Patients should not be exposed to unnecessary radiation to identify apical pathosis, which this study achieved by employing CBCT records obtained for other medical/dental reasons. The CBCT scans were obtained from one hospital, which avoided possible biases such as variances in exposure duration and setting. Study limitations include its attempt to analyze the untreated canals’ correlation with AP in a restricted number of towns surrounding educational dental hospitals. The reviewer individuals come from various locations, but they do not cover the entire Saudi population. The patient’s immune status is an important factor that was not recorded in the dental files. Information on AP status when therapy is performed was anonymous, which implies that certain lesions may heal after the treatment is completed. Thus, a cause-effect relationship cannot be determined [49]. Furthermore, several factors, including the preoperative state of the tooth, asepsis, techniques, materials, and clinician skills, might influence endodontic treatment outcomes and must be considered in future studies [46,48,50-52].

## Conclusions

The prevalence of AP in endodontically treated teeth with untreated canals was high (85.4%), with the most identified untreated canals being maxillary and mandibular first molars. In previously endodontically treated teeth with missed canals, AP was highly predictable in DM patients even with well-controlled conditions. However, well-controlled HTN was not considered to be a major factor in developing AP even when the treated tooth contained a missed canal. To optimize the knowledge of the root canal system before initiating treatment, all possible measures, including CBCT and a dental operational microscope, should be employed. More investigations are needed to provide a comprehensive evaluation and diagnosis of untreated canals associated with AP by histological, radiographic, and clinical evaluation.
